# Peter Harper

**DOI:** 10.1038/s41431-021-00864-3

**Published:** 2021-03-25

**Authors:** Julian Sampson, Angus Clarke

**Affiliations:** Institute of Medical Genetics, Cardiff University and University Hospital of Wales, Cardiff, Wales UK

**Keywords:** Genetics research, Genetic testing


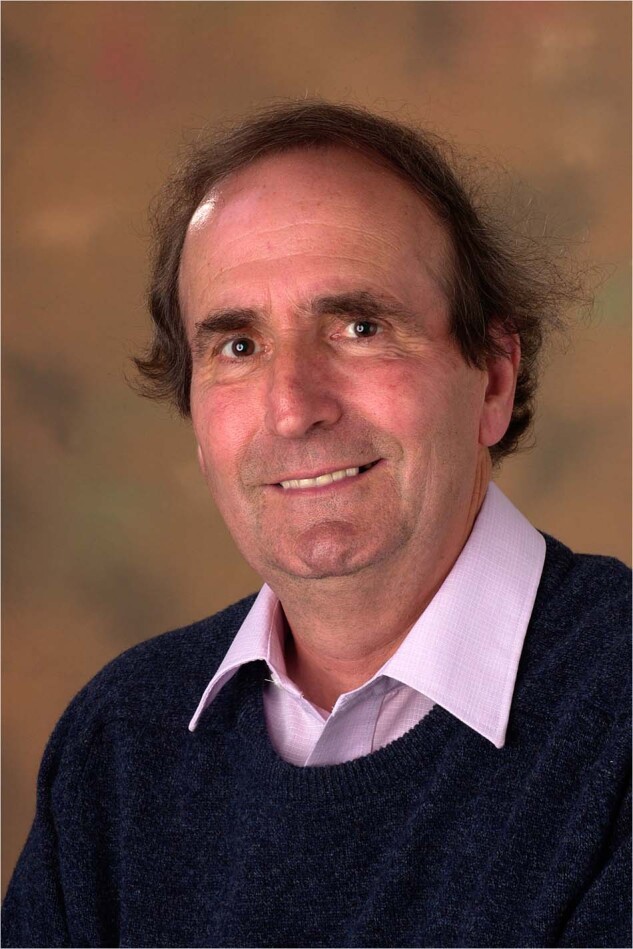


Peter Harper, Emeritus Professor at the School of Medicine in Cardiff University, Wales, an outstanding and internationally respected clinical geneticist, has died at the age of 81. He had worked in Cardiff since 1971 and retired in 2004.

Peter was born and brought up in Barnstaple, a small country town in Devon, England, where his father was a GP. His mother was a scholar of French with a first-class degree from Oxford and a Doctorate from the Sorbonne but left this behind in the move to Barnstaple. Peter grew up with a deep love of natural history and the Devon countryside. He considered a career in biology but instead chose to follow his father into medicine. He studied pre-clinical medicine at Oxford University from 1957 where he attended lectures in genetics and biology at the Department of Zoology in addition to his medical studies. After completing his clinical training in St Thomas’s Hospital (London) in 1964, he was determined to combine genetics and medicine in his future career. He took jobs in paediatrics and internal medicine before moving in 1967 to Liverpool, to work for 2 years with Prof. Cyril Clarke (later Sir Cyril, president of the Royal College of Physicians of London). Cyril had developed a strong interest in genetics through his research on the swallow-tailed butterfly, and he had recently established the Nuffield Unit of Medical Genetics at the medical school. In Liverpool Peter worked on inherited oesophageal cancer at the medical school and on insect genetics at the Zoology Department. He then moved, with his wife Elaine, to Baltimore, where Peter became a research fellow at Johns Hopkins with Victor McKusick and completed a doctorate on myotonic dystrophy. He maintained a strong interest in this disease throughout his career.

Returning to the UK in 1971, Peter was appointed as clinical lecturer in the Department of Medicine at the University of Wales College of Medicine (then a separate institution from Cardiff University). He developed links with biochemists, pathologists and others, and brought them together to establish a Department of Medical Genetics. This integrated NHS clinical genetics service delivery with laboratory diagnostics and research, while Peter also retained a role in acute general medicine. The clinical team started to grow, with services established to cover the whole of Wales, and Peter’s research interests focused particularly on the muscular dystrophies and Huntington’s disease. Cardiff soon became a renowned centre for both.

The Department of Medical Genetics was consolidated in 1987 when the Institute of Medical Genetics was opened on the University Hospital of Wales campus. This was an integrated academic and NHS centre that housed genetics outpatient clinics, clinicians and genetic counsellors, NHS and university molecular genetics teams, cytogeneticists, laboratories for newborn biochemical screening and foetal pathology, experts in computer programming and mathematical genetics, social scientists, psychiatrists and psychologists. The diversity of professionals created a unique atmosphere in which many different skills and perspectives were brought to bear on inherited conditions. Peter’s long-term projects to identify the genes for myotonic dystrophy and Huntington’s disease were bought to a successful conclusion through international collaboration. Under his leadership, an integrated and holistic approach was developed in tackling human genetic disorders. While genes were mapped and isolated in one laboratory and applied to diagnostics in another, approaches to genetic counselling and family support were developed and applied to the benefit of the families who had contributed to the gene mapping studies.

Peter also addressed questions of ethics and policy, within the UK and also internationally, through the European Society of Human Genetics and international patient organisations. Debates to which he made major contributions included those on the use of genetic testing in insurance, the genetic testing of children and the genetic counselling support for predictive genetic testing. Throughout these endeavours, the views of patients and families were always valued and Peter worked closely with organisations such as the Myotonic Dystrophy Support Group and the Huntington’s Disease Association. A steady stream of scientists, clinicians and genetic counsellors spent time training at the Institute and were also welcomed into Peter and Elaine’s home, often becoming friends as well as colleagues. Many went on to become leaders in their professions, working in other centres and seeding aspects of Peter’s holistic approach internationally as well as within the UK.

As the Medical School in Cardiff merged with Cardiff University and the demarcation between academic work and service delivery was strengthened by institutional pressures, Peter managed to ‘ride both horses’. He often resisted unhelpful interventions from outside, while ensuring that both the academic and the service delivery aspects of the Institute of Medical Genetics were able to flourish.

Peter’s personal style was informal, generous and encouraging. He helped numerous junior colleagues to develop their potential to an extent they had not envisaged. When problems arose and events proved difficult, Peter’s response was invariably to reframe the problem as an opportunity, both to learn and to publish. His writing was always clear and accessible. In addition to numerous papers he authored a number of books, some aimed at professionals, others for patients. His “Practical Genetic Counselling” was hugely successful, being translated into numerous languages and, after seven previous editions, recently being updated as an eighth edition by one of us with the support of the other.

As with everything else, Peter made clear and helpful decisions about stepping back first from his role as head of the clinical service and then as head of the academic department. He developed a long-term project to record the history of medical genetics (the Genetics and Medicine Historical Network), primarily through interviews with those who had been involved, many in advanced old age, and the archiving of their documents. This, along with walking the beautiful coast of Wales, occupied much of Peter’s time until the end of his life. He would set off, with audio-recorder in one hand and binoculars in the other, to interview retired friends and colleagues abroad or to attend a symposium (he was a sought after invited speaker to the end) while combining his academic activities with excursions to view the local avian exotica. His historical endeavours also included careful documentation of past and contemporary abuses of genetics in Europe, America, Russia and China. This important work, particularly recording the personal testimonies and reflections of elderly clinicians and scientists, required trust and understanding and was something that only someone of Peter’s stature could have accomplished. He reported these efforts in his books, “A Short History of Medical Genetics” (2008) and “Evolution of Medical Genetics—A British Perspective” (2020) and additional material, including many interviews, can be accessed online at: www.genmedhist.org.

Peter Harper was active in professional societies and networks nationally and internationally. Within the UK, he was prominent in the Clinical Genetics Society, the British Society for Human Genetics (now the British Society for Genetic Medicine) and the Royal College of Physicians. Internationally, he engaged with the European Society of Human Genetics (he was the first ESHG Award Laureate in 2003), the American College of Medical Genetics (which awarded him its lifetime achievement award) and the American Society of Human Genetics. He was Editor of the *Journal of Medical Genetics* for 10 years (1986–1996), a member of the Human Genetics Commission and the Nuffield Council for Bioethics. He was awarded a CBE (1994) and a Knighthood (2004) for services to medicine and to medical genetics.

Peter always prioritised spending time with his family as a husband, father and grandfather. He particularly enjoyed sharing his passion and his extensive knowledge of nature and wildlife with the family and made visits to his eldest son and his family in Australia each year. Peter is survived by his wife Elaine (m. 1968) and their children, Mathew, Emma, Nicholas, Katy and Lucy.

